# Identification and characterization of regulatory network components for anthocyanin synthesis in barley aleurone

**DOI:** 10.1186/s12870-017-1122-3

**Published:** 2017-11-14

**Authors:** Ksenia V. Strygina, Andreas Börner, Elena K. Khlestkina

**Affiliations:** 1 Institute of Cytology and Genetics, Siberian Branch of the Russian Academy of Sciences, Lavrentjeva ave. 10, Novosibirsk, 630090 Russia; 2 Leibniz Institute of Plant Genetics and Crop Plant Research (IPK), Corrensstr. 3, 06466 Stadt Seeland, OT Gatersleben Germany; 3 Novosibirsk State University, Pirogova str., 1, Novosibirsk, 630090 Russia

**Keywords:** bHLH, Cytochrome P450, Flavonoid biosynthesis, Gene duplication, *Hordeum*, MBW, MYB, MYC, Transcription factor, WD40

## Abstract

**Background:**

Among natural populations, there are different colours of barley (*Hordeum vulgare* L.). The colour of barley grains is directly related to the accumulation of different pigments in the aleurone layer, pericarp and lemma. Blue grain colour is due to the accumulation of anthocyanins in the aleurone layer, which is dependent on the presence of five *Blx* genes that are not sequenced yet (*Blx1*, *Blx3* and *Blx4* genes clustering on chromosome 4HL and *Blx2* and *Blx5* on 7HL). Due to the health benefits of anthocyanins, blue-grained barley can be considered as a source of dietary food. The goal of the current study was to identify and characterize components of the anthocyanin synthesis regulatory network for the aleurone layer in barley.

**Results:**

The candidate genes for components of the regulatory complex MBW (consisting of transcription factors **M**YB, **b**HLH/MYC and **W**D40) for anthocyanin synthesis in barley aleurone were identified. These genes were designated *HvMyc2* (4HL), *HvMpc2* (4HL), and *HvWD40* (6HL). *HvMyc2* was expressed in aleurone cells only. A loss-of-function (frame shift) mutation in *HvMyc2* of non-coloured compared to blue-grained barley was revealed. Unlike aleurone-specific *HvMyc2*, the *HvMpc2* gene was expressed in different tissues; however, its activity was not detected in non-coloured aleurone in contrast to a coloured aleurone, and allele-specific mutations in its promoter region were found. The single-copy gene *HvWD40*, which encodes the required component of the regulatory MBW complex, was expressed constantly in coloured and non-coloured tissues and had no allelic differences. *HvMyc2* and *HvMpc2* were genetically mapped using allele-specific developed CAPS markers developed. *HvMyc2* was mapped in position between SSR loci *XGBS0875-4H* (3.4 cM distal) and *XGBM1048-4H* (3.4 cM proximal) matching the region chromosome 4HL where the *Blx-*cluster was found. In this position, one of the anthocyanin biosynthesis structural genes (*HvF3’5’H*) was also mapped using an allele-specific CAPS-marker developed in the current study.

**Conclusions:**

The genes involved in anthocyanin synthesis in the barley aleurone layer were identified and characterized, including components of the regulatory complex MBW, from which the MYC-encoding gene (*HvMyc2*) appeared to be the main factor underlying variation of barley by aleurone colour.

**Electronic supplementary material:**

The online version of this article (10.1186/s12870-017-1122-3) contains supplementary material, which is available to authorized users.

## Background

Flavonoids are natural biologically active compounds produced by plants. Flavonoid pigment anthocyanins are known for their plant protective functions [1, 2] and human health benefits [3, 4].

**Fig. 1:**
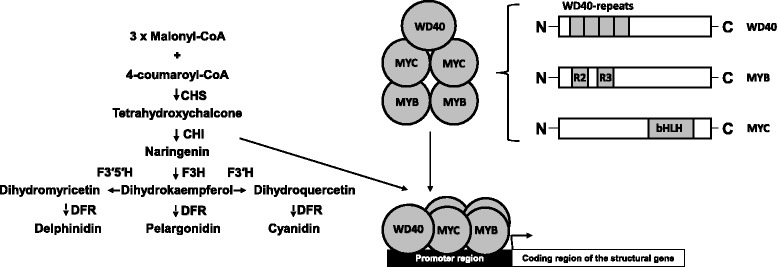
Scheme of anthocyanin biosynthesis (modified from [42]). MYB, MYC, WD40 – transcription factors. WD40-repeats, R2R3, bHLH – their functional domains. CHI – chalcone-flavanone isomerase; CHS – chalcone synthase; DFR – dihydroflavonol 4-reductase; F3H – flavanone 3-hydroxylase; F3’H – flavonoid 3′-hydroxylase; F3’5’H – flavonoid 3′,5′-hydroxylase

Diverse coloration patterns in plants are achieved through a wide variety of regulatory factors involved in the biosynthesis of flavonoid pigments. The activation of flavonoid biosynthesis occurs with the help of the MBW complex, which is composed of three types of transcription factors, **M**YB, **b**HLH/MYC and **W**D40 [5–7] (Fig. 1). These regulatory elements activate the structural genes encoding enzymes involved in the biosynthesis of flavonoids, providing tissue-specific accumulation of the pigment.

Barley (*Hordeum vulgare* L.) is an important agricultural crop. In natural populations, barley plants with different types of grain coloration are described. Purple, yellow and blue types of pigmentation are associated with the accumulation of flavonoid pigments in diverse layers of the grains. The purple colour of grain pericarp depends on the *HvAnt2* gene. It is located on the long arm of chromosome 2H (2HL) and encodes the bHLH/MYC protein [8–10], which together with the MYB factor (putatively encoded by the *HvMpc1*/*HvAnt1* gene (7HS) [11, 12]) activates the structural genes. The appearance of proanthocyanidin pigmentation in the barley seed coat is associated with expression of the *HvAnt28* gene (3HL), encoding the MYB-type factor [13, 14]. The blue colour of the aleurone layer depends on the presence of five complementary genes that have not been sequenced yet: *Blx1*, *Blx2*, *Blx3*, *Blx4* and *Blx5*. Three of these genes (*Blx1*, *Blx3*, *Blx4*) are closely linked to each other and were mapped to chromosome 4HL. *Blx2* and *Blx5* are located at chromosome 7HL [15]. A change of aleurone colour from blue to pink (red) occurs when complementary dominant alleles are present at the *Blx1*, *Blx2*, *Blx3*, and *Blx5* loci but not at *Blx4* [15].

MYB and MYC factors regulate anthocyanin synthesis in aleurone and their relation to the *Blx* genes are not yet known. The WD40 component of the barley MBW regulatory complex for anthocyanin synthesis has also not been identified and studied yet.

In the current study, we checked a database for barley sequences that have not been annotated to find and analyse genes encoding transcription factors MYC, MYB and WD40, which are related to anthocyanin synthesis in the aleurone layer, as well as *F3′5′H* – a putative candidate gene for *Blx4*.

## Results

### Identification, sequencing and study of the structural organization of the genes regulating anthocyanin synthesis in barley aleurone

#### bHLH/MYC

We found one copy of the MYC-encoding *HvAnt2* gene (GenBank: KX035100) located on the long arm of chromosome 4H (Table 1). The predicted coding sequence (1683 bp in length) of the gene designated *HvMyc2* shares 70.8% identity with *HvAnt2.*

**Table 1:** The anthocyanin synthesis regulatory genes annotated for the first time in the current study

Gene name	Protein type	CDS length, bp	Chromosome	Cultivar	Contig from IPK Barley BLAST Server	Exons
*HvMyc2*	MYC/bHLH	1683	4HL	Bowman	106,753	e1-e5
					10,625	e6-e8
				Morex	1,563,805	e1-e5
					442,143	e6-e8
				Barke	430,151	e1-e2
					55,550	e3-e5
					2,789,433	e6-e8
*HvMpc2*	MYB	711	4HL	Bowman	110,138	e1-e2
				Morex	317,820	e1-e2
				Barke	401,169	e1-partially e2
					395,048	partially e2
*HvWD40*	WD40	1071	6HL	Bowman	849,119	e1
				Morex	39,083	e1
*HvF3’5’H*	Cytochrome P450	1440	4HL	Bowman	855,926	e1-e3
				Morex	1,575,914	e1-e3

**Fig. 2:**
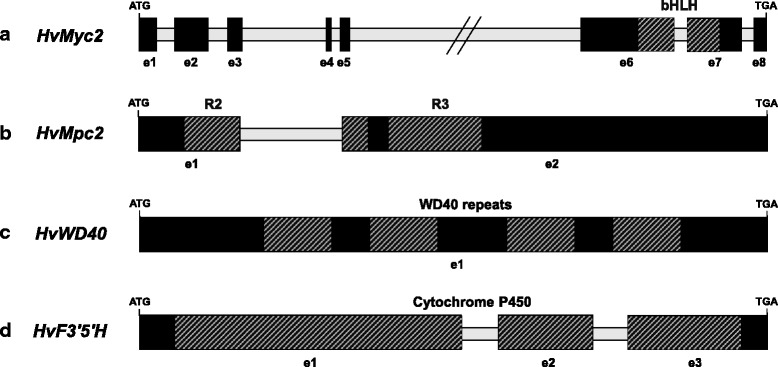
Structural organization of the genes: **a** *HvMyc2*; **b** *HvMpc2*; **c** *HvWD40*; **d** *HvF3’5’H.* striped boxes indicate functional domains: Myc-type basic helix-loop-helix (bHLH) domain (InterPro: IPR011598); Myb-like DNA-binding domain (InterPro: PF00249); WD40 repeats (InterPro: SM00320) and WD domain, G-beta repeat (InterPro: PF00400); Cytochrome P450, E-class, group I (InterPro: IPR002401)

**Fig. 3:**
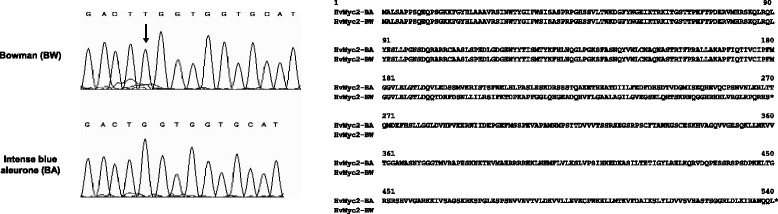
The single nucleotide insertion in the gene *HvMyc2* and alignment of predicted amino acid sequences of the *HvMyc2-BA* and *HvMyc2-BW* alleles

The complete coding sequence of *HvMyc2* was obtained. It consists of eight exons. The 6th and 7th exons contain the conservative MYC-type bHLH domain required for gene activation through binding with DNA and protein (Fig. 2). Comparison of *HvMyc2* sequences of BW and BA near-isogenic lines differing by the *Ba* gene allelic state, showed several synonymous single nucleotide substitutions and one single nucleotide deletion (58 bp upstream bHLH-encoding motif), resulting in a frame-shift in uncoloured BW (Fig. 3). Sequencing of the partial *HvMyc2* gene in the parents of the mapping population (DOM and REC) revealed the same loss-of-function mutation in the uncoloured REC parent. We developed a CAPS marker specific for the functional *HvMyc2-BA* allele and used it for amplification of DNA from different barley varieties having a blue or uncoloured aleurone colour. The *HvMyc2-BA* was present in all blue-grained barleys and in two genotypes with uncoloured aleurone (OWB_03 and OWB_28) (Additional file 1). The presence of the *HvMyc2-BA* allele in some samples lacking anthocyanin synthesis in aleurone can be explained either by other mutations in *HvMyc2* or by putative loss-of-function mutations in other anthocyanin biosynthesis genes.

#### MYB

The full-length gene sequence (861 bp) with 69.0% identity to the R2R3 MYB-encoding gene *HvAnt1* (GenBank:KP265977) was found on the 4HL chromosome (Table 1). The gene was designated *HvMpc2.* The *HvMpc2* gene was re-sequenced in BW, BA, DOM and REC. The gene consisted of two exons (Fig. 2) and carried R2- and R3-motifs required for polyphenol biosynthesis. The coding sequences of coloured (BA, DOM) and uncoloured (BW, REC) genotypes contained only synonymous single nucleotide substitutions. Some indels were revealed in the promoter region (Additional file 2).

#### WD40

After BLAST analysis, using sequences of the WD40-encoding genes of maize (GenBank: AY115485 [16]) and sorghum (GenBank: JX122967 [17]), we found the orthologous gene (full-length sequence 1071 bp in length) on barley chromosome 6HL (with a level of identity 87.0% compared to maize *ZmPAC1* and 87.8% with sorghum *SbTan1*) (Table 1). The gene was designated *HvWD40*. *HvWD40* was re-sequenced in BW, BA, DOM and REC. The gene lacked introns and contained four WD40 repeats with Trp-Asp (WD) doublet residues at the C-terminus (Fig. 2). Four genotypes that were sequenced differed from each other by synonymous single nucleotide substitutions only.

#### F3’5’H

Our search in databases was based on sequences of *F3’5’H* genes of dicots: grape (GenBank: NM_001281235), lycium (GenBank: KC161969), soybean (GenBank: EF174665), blackcurrant (GenBank: KC493688), balloon flower (GenBank: JQ403611), cyclamen (GenBank: GQ891056) (several sequences for BLAST search were taken for cross-validation). The sequence (full-length 1689 bp in length) with the highest identity was found on chromosome 4HL, where *Blx4* is located (Table 1). This gene was designated *HvF3’5’H*. The coding region of *HvF3’5’H* was separated into three exons (Fig. 2). It contained a cytochrome P450 motif (InterPro: IPR002401). BW, BA, DOM and REC differed from each other by synonymous single nucleotide substitutions only.

### RT-PCR analysis of transcriptional activity of the studied genes

**Fig. 4:**
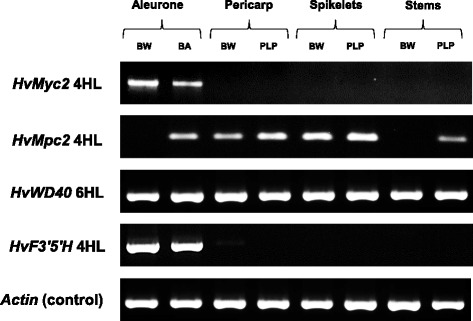
Expression of the *HvMyc2*, *HvMpc2*, *HvWD40* and *HvF3’5’H* genes in the aleurone layer, pericarp, lemmas (with developing spikelets) and stems (with leaf sheaths) of NILs differing by coloration

Tissue-specific expression of *HvMyc2* and *HvF3’5’H* genes was observed in the aleurone layer, both in coloured and uncoloured near-isogenic lines (Fig. 4). They were not transcribed in the pericarp nor in the lemma (with developing spikelets) and stem (with leaf sheath). Unlike aleurone-specific *HvMyc2* and *HvF3’5’H*, the *HvMpc2* gene was expressed in all mentioned tissues; however, its activity was not detected in non-coloured aleurone in contrast to coloured aleurone. *HvWD40* transcripts were observed in all analysed cDNA samples (Fig. 4).

### Molecular mapping

**Fig. 5:**
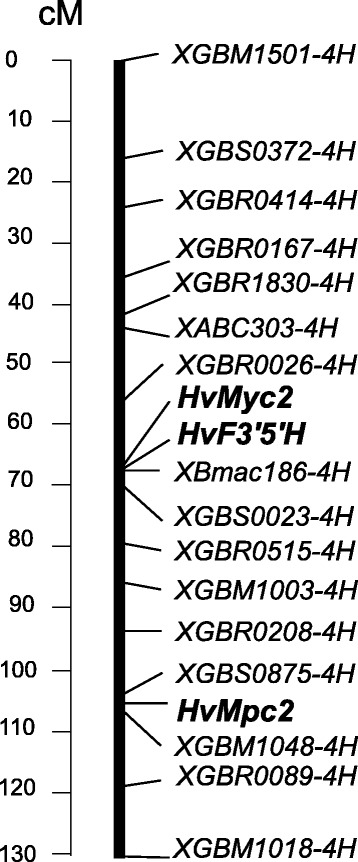
Molecular-genetic mapping of the *HvMyc2*, *HvF3’5’H* and *HvMpc2* genes on barley chromosome 4H

For molecular mapping, we selected the Oregon Wolfe Barley (OWB) population. This population of doubled-haploid lines was segregated for anthocyanin grain coloration and for the *HvMyc2-BA* specific CAPS marker (Table 2). Forty-four lines carried *HvMyc2-BA*, while 48 lines lacked it. The segregation ratio matched the expected 1:1 (χ^2^ = 0.17; *P* > 0.50). We used *HvMyc2-BA* genotyping data together with available SSR- and RFLP-loci data for linkage analysis. The gene *HvMyc2* was closely linked to the SSR locus *XBmac186-4H*. Similarly, for mapping *HvMpc2* and *HvF3’5’H,* CAPS-markers were developed (based on single nucleotide polymorphisms between DOM and REC) (Fig. 3). The segregation ratio for *HvMpc2* (44:48) and *HvF3’5’H* (44:48) matched the expected 1:1 (χ^2^ = 0,17; P > 0.50 for *HvMpc2*; χ^2^ = 0,17; P > 0.50 for *HvF3’5’H*). The *HvF3’5’H* gene mapped closely to *XBmac186-4H* and *HvMyc2*, while *HvMpc2* mapped between SSR loci *XGBS0875-4H* (3.4 cM distal) and *XGBM1048-4H* (3.4 cM proximal) (Fig. 5)*.*

**Table 2:**
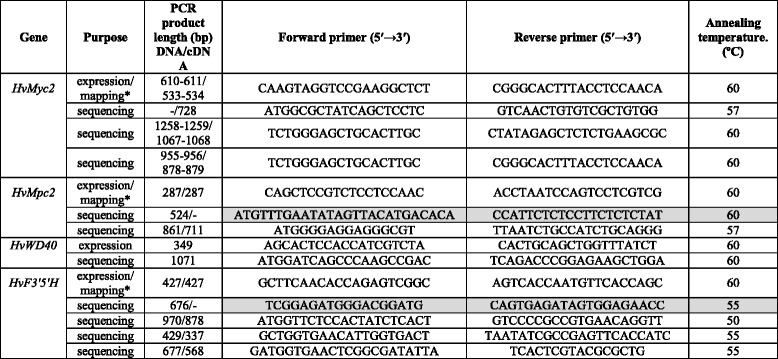
Gene-specific primers used for amplification of barley DNA. For primer design, the contigs sequences mentioned in Table 1 were used. Primer pairs used for promoter region sequencing are shaded by a grey colour

*For molecular mapping, amplified fragments of the mapping population individuals were digested with restriction endonucleases *Bse1* I, *Hga* I and *EcoR* I respectively (CAPS –analysis)

## Discussion

Duplications are the main source of new genes in genomes – approximately 90% of genes found in eukaryotes are the result of duplications [18, 19]. The retention of transcription factors after gene duplication suggest that the expansion of transcription factor (TF) families may provide adaptive benefits [20]. The proteins of the MYB family belong to the most numerous class, and the bHLH proteins are the second largest class of transcription factor families among the plant classifications. The proteins bHLH and MYB interact with the protein WD40, forming a highly dynamic complex MYB/bHLH/WD40 (MBW). These complexes regulate various cellular processes such as responses to the biotic and abiotic stresses, formation of root hairs and trichomes, and synthesis of phenolic compounds including flavonoids [5–7]. In barley, not all of the MYB and MYC factors, which are involved in flavonoid biosynthesis, have been identified and described. The data on structural and functional organization and chromosome localization of barley WD40-encoding genes is missing. In the current study, we identified and characterized components of the MBW complex for anthocyanin synthesis in aleurone cells of barley grain.

The bHLH/MYC encoding gene identified in the current study (*HvMyc2* on chromosome 4HL) appeared to be a paralogous copy of the *HvAnt2* gene conferring purple pericarp colour and it was located on chromosome 2H. An earlier study aimed to identify copies of the gene *TaMyc1,* which regulates anthocyanin synthesis in wheat pericarp. The study discovered 11 copies of homoeologous group 2 and homoeologous group 4 chromosomes [21]. It was concluded that the first duplication of the *Myc* gene occurred in the common ancestor of the Triticeae tribe [21]. *HvMyc2*, which was identified in the current study, likely originated from the duplicated *Myc* gene on the ancestor’s chromosome 4 and is likely an orthologue of some of the *TaMyc1* copies localized on chromosomes 4AL, 4BL and 4DL. Among wheat species, the *Ba* gene for blue aleurone was found in *Triticum boeoticum* only [22]. Bread wheat (*T. aestivum*) may have blue aleurone colour only due to substitution of one of the homoeologous group 4 chromosomes by chromosome 4Ag of *Agropyron* [22]. In barley, chromosome 4H is known to be responsible for blue aleurone control [15, 23] and carries an orthologue of the *Ba* genes of *T. boeoticum* and *Agropyron*. Our findings suggest that the MYC-encoding gene (*HvMyc2*) is the main component of the regulatory network underlying barley variation by aleurone colour. First, we observed specific expression of this gene in aleurone only, while other candidate genes did not show such specificity (Fig. 4). Second, we have found a loss-of-function (reading frame shift; Fig. 3) mutation in most barley samples having uncoloured aleurone (Additional file 1). Furthermore, comparison of the *HvMyc2* precise position on chromosome 4HL with that of the *Blx1*/*Blx3*/*Blx4* cluster [15, 23, 24] resulted in a conclusion about their colocalization. We suggest *HvMyc2* is a candidate gene for *Blx1* or *Blx3*, while *HvF3’5’H* was proved to be *Blx4*. It is known that loss-of-function mutations in the gene encoding the F3’5’H enzyme result in lack of the ‘blue’ fraction of anthocyanins [25] and that mutation of *Blx4* results in a change of aleurone colour from blue to pink (red) [15]. Furthermore, *HvF3’5’H* is colocalized with the *Blx4* gene (Fig. 5, [24]).

Like *HvMyc2*, the *HvF3’5’H* has aleurone specific expression (Fig. 4). Tissue-specific expression of a structural gene may indicate the presence of a potential duplicated copy. For example, two paralogous copies of the flavonoid biosynthesis *F3 h* gene occur in some Triticeae species, with one copy specifically expressed in roots and the second copy active in other parts of the plant [26]. We suggest that the barley genome should contain another *F3′5′H* copy due to delphinidin derivate accumulation, not only in the aleurone layer but also, for example, in pericarps and stems [8, 12]. The presence of two *F3′5′H* copies is not rare within plant species.

The MYB-encoding gene *HvMpc2* identified in the current study on chromosome 4HL appeared to be located distal to the *Blx1*/*Blx3*/*Blx4* cluster. This gene could be an orthologue of some of the wheat *TaPL1* gene copies on 4BL and 4DL chromosomes [27]. *HvMpc2* is expressed in several parts of barley plants and shows no specificity to aleurone. Nevertheless, the product of the *HvMpc2* gene is assumed to be a part of a regulatory anthocyanin synthesis BMW complex in aleurone, since *HvMpc2* encodes a MYB-like transcriptional factor that shows high similarity to HvMpc1 (which regulates biosynthesis of anthocyanins in the purple leaf sheath [12]) and because *HvMpc2* expression correlates with aleurone colour (Fig. 4). Location of *HvMpc1* (7HS) is distinct from that of *Blx2* and *Blx5* on 7HL (*Blx2* and *Blx5* products remain unknown; probably they encode enzymes necessary for anthocyanin molecule modifications). Thus, both MYB-encoding anthocyanin regulatory genes in barley (*HvMpc1* and *HvMpc2*) do not colocalize with *Blx* genes and are not related to variation in blue aleurone colour of barley. Variation of anthocyanin pigmentation in different parts of plants is usually caused by mutations of MYB-encoding genes, while the MYC-encoding partner is more conservative. The leading role of the MYB-encoding gene variability in phenotypic variation was observed in wheat coleoptiles, stems, leaf sheaths, leaf blades and anthers [28, 29] and barley leaf sheaths [12]. In the case of pericarp coloration, both MYB- and MYC-encoding regulatory genes contribute to phenotypic variation [30, 31], while in the case of aleurone coloration, the Myc-gene contribution is essential (current study).

In addition, we identified the WD40-coding gene, which was designated *HvWD40* (6HL). The single-copy gene *HvWD40* that encodes the required component of the regulatory MBW complex was expressed constantly in coloured and non-coloured tissues, and had no allelic differences. Proteins of this class are involved in a variety of cellular processes, which is probably the reason why they are specified by high conservatism [32]. *WD40* genes of other plant species (potato, for example) share a similar expression profile, which does not correlate with tissue-specificity or the intensity of tissue colour.

We assume that *HvWD40* together with *HvMpc2* and *HvMyc2* forms the MBW regulatory complex, which is necessary for activation of the structural anthocyanin biosynthesis genes in aleurone, while with *HvAnt1* and *HvAnt2,* it forms the MBW complex that is necessary for regulation of anthocyanin synthesis in pericarps.

## Conclusions

Genes involved in anthocyanin synthesis in the barley aleurone layer were identified and characterized, including components of the MBW regulatory complex, from which the MYC-encoding gene (*HvMyc2*) appeared to be the main factor underlying variation of barley aleurone colour.

## Methods

### Plant material

Two parental lines (DOM and REC) and 92 plants from the barley mapping population Oregon Wolfe Barleys (OWB) [33], three Bowman’s near-isogenic lines (NILs) (Table 3), nine cultivars from the ICG collection “GenAgro” (Novosibirsk, Russia) and two accessions from IPK GenBank (Gaterslebendeclar, Germany) were screened for the presence of the *HvMyc2-BA* allele (Additional file 1). The three NILs were exploited for gene expression analysis. DOM, REC, BW and BA were also used for sequencing. The plants were grown in ICG Greenhouse Core Facilities (Novosibirsk, Russia) under a 12 h photoperiod at 20–25 °C.

**Table 3:** *Hordeum vulgare* ‘Bowman’ near-isogenic lines (NILs) that were used and their phenotypic characteristics

Line designation	NGB* ID	Phenotype of analysed tissue			
		Aleurone	Pericarp	Lemma	First leaf sheath
BW (Bowman)	NGB22812	uncoloured	uncoloured	uncoloured	uncoloured
BA (Blue aleurone)	NGB20651	blue	uncoloured	uncoloured	purple
PLP (Purple lemma and pericarp)	NGB22213	uncoloured	purple	purple	purple

*NGB – Nordic GenBank

### Gene identification and in silico analysis

A database search for homologous sequences was carried out for not annotated barley sequences deposited at the IPK Barley BLAST Server (http://webblast.ipk-gatersleben.de/barley_ibsc/) using software provided at this Server [34]. Annotation of the detected sequences was performed using the FGENESH+ program [35] and confirmed by cDNAs sequencing. Alignment of nucleotide and amino acid sequences was made using the MULTALIN v5.4.1 program [36]. Barley genes *HvAnt2* (GenBank: KX035100) and *HvAnt1* (GenBank: KP265977) were used to identify *Myc*-like and *Myb*-like sequences, respectively. The search for the WD40-coding gene was made with the maize *ZmPAC1* (GenBank: AY115485) and the sorghum *SbTan1* (GenBank: JX122967) genes. For identification of the gene encoding F3′5′H a search was done using known *F3′5′H* gene sequences of dicot plants species: *VvF3’5’H* (GenBank: NM_001281235), *LrF3’5’H* (GenBank: KC161969), *GmW1* (GenBank: EF174665), *RnF35H* (GenBank: KC493688), *PgF3’5’H* (GenBank: JQ403611), and *CpF3’5’H* (GenBank: GQ891056). Amino acid sequences were predicted using InterPro [37]. The exon-intronic structure of the genes was predicted with FGENESH+ software [35] using polypeptide sequences of homologous genes *HvAnt2*, *HvAnt1*, *ZmPAC1* and *VvF3’5’H.*

### DNA and RNA extraction, cDNA synthesis

Total genomic DNA was extracted from fresh leaves of plants following a procedure described earlier [38]. Pericarps and aleurones for RNA extraction were scalpeled from grains at early dough stage maturity (BBCH code 83) for BW and BA lines. RNAs from pericarp as well as from lemmas (with developing spikelets; collected at the end of flowering; BBCH code 69) and stems (with leaf sheaths; collected at the same stage) were extracted applying a ZR Plant RNA MiniPrep™ (Zymo Research, USA). RNAs from aleurone layer samples were extracted using a RNeasy Mini Kit (QIAGEN, Germany). All isolated RNAs were treated with RNase-free DNase set (QIAGEN, Germany). Total RNA was converted to single-stranded cDNA in a 20-μL reaction from a template consisting of 0.2 μg of total RNA using a RevertAid First Strand cDNA Synthesis Kit (Thermo Fisher Scientific Inc., USA).

### PCR, restriction and sequence analysis

Amplification of gDNA and cDNA was made in 20 μL PCRs. Reaction mixtures contained 50–100 ng of genomic template DNA, 1 ng of each of primer, 0.25 mM of each dNTP, 1× reaction buffer (67 mM TrisHCl, pH 8.8; 2 mM MgCl_2_; 18 mM (NH_4_)_2_SO_4_; 0.01% Tween 20) and 1 U Taq polymerase. DNA templates were amplified with initial denaturation at 94 °C for 2 min, 35 cycles were run at 94 °C for 1 min, 50-60 °C for 1 min, and 72 °C for 0.5-2 min, followed by a final extension at 72 °C for 5 min. Primer design was carried out using the OLIGO7 program. PCR products were separated on agarose gels, stained with ethidium bromide and visualized under UV light. The amplified fragments were purified from an agarose gel using a DNA Clean kit (Cytokine, St. Petersburg, Russia). To discriminate different alleles of the *HvMyc2*, *HvMpc2* and *HvF3’5’H* loci, we developed CAPS markers. Corresponding PCR products (Table 2) were digested with restriction endonucleases *Bse1* I, *Hga* I and *EcoR* I, respectively, followed by separation of DNA fragments in a 2-5% high resolution agarose gel (HydraGene Co., China) (Additional file 3). DNA sequencing was performed using the SB RAS Genomics core facilities (Novosibirsk, Russia). The full-length gene sequences were re-constructed from a series of the overlapping amplicons (Table 1). All obtained sequences were deposited in GenBank (NCBI).

### Genetic mapping

Identified loci were mapped relative to RFLP and SSR loci of the Oregon Wolfe Barleys (OWBs) mapping population [33, 39]. The genes *HvMyc2*, *HvF3’5’H* and *HvMpc2* were mapped using the gene-specific CAPS marker developed in the current study. Linkage maps were constructed with MAPMAKER 2.0 [40] using the Kosambi function [41].

## Additional files

Additional file 1: The presence of the *HvMyc2-BA* allele in barley Bowman NILs (1–2), parents of the mapping population used (3–4), recombinant DH lines of this population (5–96), accessions and cultivars from IPK Genbank (97–98) and ICG collection GenAgro (99–107). (PDF 149 kb)

Additional file 2: Multiple alignment of the promoter regions of the barley *HvMpc2* gene*. (PDF 196 kb)*

Additional file 3: The results of genotyping of the mapping population (Oregon Wolfe Barleys, OWB) for molecular mapping of barley genes: A – *HvMyc2* B – *HvMpc2* C – *HvF3’5’H*. Amplified fragments of the mapping population individuals of were digested with restriction endonucleases *Bse1* I, *Hga* I and *EcoR* I respectively (CAPS–analysis). DOM and REC are the parental lines. (PDF 327 kb)
