# Supplementary material for: Identification and characterization of regulatory network components for anthocyanin synthesis in barley aleurone
Source: BMC Plant Biol. 2017 Nov 14;17(Suppl 1):184. doi: 10.1186/s12870-017-1122-3 (PMC5688479; doi:10.1186/s12870-017-1122-3)
Supplement: Supplementary file 1 — The presence of the HvMyc2-BA allele in barley Bowman NILs (1–2), parents of the mapping population used (3–4), recombinant DH lines of this population (5–96), accessions and cultivars from IPK Genbank (97–98) and ICG collection GenAgro (99–107). (PDF 149 kb) [file 12870_2017_1122_MOESM1_ESM.pdf]

Additional file 1. The presence of the *HvMyc2-BA* allele in barley Bowman NILs (1-2), parents of the mapping population used (3-4), recombinant DH lines of this population (5-96), accessions and cultivars from IPK Genbank (97-98) and ICG collection GenAgro (99-107).

No	Genotype	Grain color	The presence of the *HvMyc2-BA* allele determined by the use of allele specific DNA-marker
1.	Bowman, BW (NGB22812)	white	+
2.	Blue aleurone, BA (NGB20651)	blue	-
3.	OWB_DOM	black	+
4.	OWB_REC	white	-
5.	OWB_01	black	+
6.	OWB_02	white	-
7.	OWB_03	white	+
8.	OWB_04	black	+
9.	OWB_05	black	+
10.	OWB_06	white	-
11.	OWB_07	black	+
12.	OWB_08	blue	+
13.	OWB_09	white	-
14.	OWB_10	black	+
15.	OWB_11	brown	+
16.	OWB_13	white	-
17.	OWB_14	white	-
18.	OWB_15	brown	-
19.	OWB_16	black	-
20.	OWB_17	brown	-
21.	OWB_18	black	+
22.	OWB_19	black	-
23.	OWB_20	black	+
24.	OWB_21	black	+
25.	OWB_22	white	-
26.	OWB_23	white	-
27.	OWB_24	brown	-
28.	OWB_26	white	-
29.	OWB_27	black	+
30.	OWB_28	white	+
31.	OWB_29	blue	+
32.	OWB_30	brown	+
33.	OWB_31	black	+
34.	OWB_32	brown	-
35.	OWB_33	white	-
36.	OWB_34	white	-
37.	OWB_35	black	-

38.	OWB_36	black	-
39.	OWB_37	black	-
40.	OWB_38	black	+
41.	OWB_39	brown	+
42.	OWB_40	brown	-
43.	OWB_41	black	+
44.	OWB_42	white	-
45.	OWB_43	white	-
46.	OWB_44	blue	+
47.	OWB_45	white	-
48.	OWB_46	white	-
49.	OWB_47	brown	-
50.	OWB_48	blue	+
51.	OWB_49	blue	+
52.	OWB_50	black	-
53.	OWB_51	brown	-
54.	OWB_52	white	-
55.	OWB_53	brown	+
56.	OWB_54	blue	+
57.	OWB_55	brown	+
58.	OWB_56	blue	+
59.	OWB_57	brown	-
60.	OWB_58	blue	+
61.	OWB_59	brown	+
62.	OWB_60	brown	-
63.	OWB_61	brown	-
64.	OWB_62	brown	+
65.	OWB_63	black	-
66.	OWB_64	black	-
67.	OWB_65	black	-
68.	OWB_66	black	+
69.	OWB_67	black	-
70.	OWB_68	black	+
71.	OWB_69	black	-
72.	OWB_70	white	-
73.	OWB_71	brown	-
74.	OWB_72	blue	+
75.	OWB_73	black	-
76.	OWB_74	brown	-
77.	OWB_75	black	+
78.	OWB_76	white	-
79.	OWB_77	brown	+
80.	OWB_78	brown	-
81.	OWB_79	white	-
82.	OWB_80	brown	+
83.	OWB_81	black	+

84.	OWB_82	white	-
85.	OWB_83	brown	-
86.	OWB_84	white	-
87.	OWB_85	blue	+
88.	OWB_86	brown	+
89.	OWB_87	brown	-
90.	OWB_88	brown	-
91.	OWB_89	brown	+
92.	OWB_90	brown	+
93.	OWB_91	purple	+
94.	OWB_92	brown	+
95.	OWB_93	blue	+
96.	OWB_94	brown	-
97.	HOR 6866	blue	+
98.	HOR17020	blue	+
99.	Biom	white	-
100.	Tanay	white	-
101.	Vorsinsky	white	-
102.	Zolotnik	white	-
103.	Omskiy-1	white	-
104.	Omskiy-2	white	-
105.	Acha	white	-
106.	Signal	white	-
107.	Sobolek	white	+
