# Supplementary material for: Identification and characterization of regulatory network components for anthocyanin synthesis in barley aleurone
Source: BMC Plant Biol. 2017 Nov 14;17(Suppl 1):184. doi: 10.1186/s12870-017-1122-3 (PMC5688479; doi:10.1186/s12870-017-1122-3)
Supplement: Supplementary file 2 — Multiple alignment of the promoter regions of the barley HvMpc2 gene. (PDF 196 kb) [file 12870_2017_1122_MOESM2_ESM.pdf]

Additional file 2. Multiple alignment of the promoter regions of the barley *HvMpc2* gene.

```
 1                                                                                   90
 HvMpc2-BA   ATGTTTGAATATAGTTACATGACACATAATTCCACAACGGGTGGGATGCTCCCTCCATGCTCACACACATCTGGGATGCCCC--------
 HvMpc2-BW   ATGTTTGAATATAGTTACATGACACATAATTCCACAACGGGTGGGATGCTCCCTCCATGCTCACACACATCTGGGATGCCCCCCTTCGGT
HvMpc2-DOM   ATGTTTGAATATAGTTACATGACACATAATTCCACAACGGGTGGGATGCTCCCTCCATGCTCACACACATCTGGGATGCCCCCCTTCGGT
HvMpc2-REC   ATGTTTGAATATAGTTACATGACACATAATTCCACAACGGGTGGGATGCTCCCTCCATGCTCACACACATCTGGGATGCCCCCCTTCGGT

             91                                                                                     180
 HvMpc2-BA   -------GGTCCG---------TGGTGACACAGGTGAGCCGTCGAGCAAAAGCTACGTGCTCTGGCTTCTTTGGACTCAAAGGGCAACGA
 HvMpc2-BW   TTTGGGGGGTCCGGGTGCCTCGTGCTGACACAGGTGAGCCGTCGAGCAAAAGCTACGTGCTCTGGCTTCTTTGGACTCAAAGGGCAACGA
HvMpc2-DOM   TTTGGGGGGTCCGGGTGCCTCGTGCTGACACAGGTGAGCCGTCGAGCAAAAGCTACGTGCTCTGGCTTCTTTGGACTCAAAGGGCAACGA
HvMpc2-REC   TTTGGGGGGTCCGGGTGCCTCGTGCTGACACAGGTGAGCCGTCGAGCAAAAGCTACGTGCTCTGGCTTCTTTGGACTCAAAGGGCAACGA

             181                                                                                    270
 HvMpc2-BA   TCCTTTTCAGTTGTCAAAAGAAATATGACATAGTACATCTCACCTGCCCGCGATCTTGGACGGCGAAGAAATGGCCGCGCTGTTCATTGG
 HvMpc2-BW   TCCTTTTCAGTTGTCAAAAGAAATACGACATAGTACATCTCACCTGCCGGCGATCTTGGACGGCGAAGAAATGGCCGCGCTGTTCATTGG
HvMpc2-DOM   TCCTTTTCAGTTGTCAAAAGAAATACGACATAGTACATCTCACCTGCCGGCGATCTTGGACGGCGAAGAAATGGCCGCGCTGTTCATTGG
HvMpc2-REC   TCCTTTTCAGTTGTCAAAAGAAATACGACATAGTACATCTCACCTGCCGGCGATCTTGGACGGCGAAGAAATGGCCGCGCTGTTCATTGG

             271                                                                                    360
 HvMpc2-BA   ACAAATGGAGCCTTCCTAGCCTTTTGGTCCATCTAACTCTGAGATAAGTGTCGCTCACGATGACTGTGGATTGAAGGTGCAAAGCAGGTC
 HvMpc2-BW   ACAAATGGAGCCTTCCTAGCCTTTTGGTCCATCTAACTCTGAGATAAGTGTCGCTCACGATGACTGTGGATTGAAGGTGCAAAGCAGGTC
HvMpc2-DOM   ACAAATGGAGCCTTCCTAGCCTTTTGGTCCATCTAACTCTGAGATAAGTGTCGCTCACGATGACTGTGGATTGAAGGTGCAAAGCAGGTC
HvMpc2-REC   ACAAATGGAGTCTTCCTAGCCTTTTGGTCCATCTAACTCTGAGATAAGTGTCGCTCACGATGACTGTGGATTGAAGGTGCAAAGCAGGTC

             361                                                                                    450
 HvMpc2-BA   CACGTAGACGCGCTACTGTACAATGCTAAACGGGTCCACGTGGACGCGCTGATGACTAAATACAG-AACAACCCTGCTGCCCAAAGATGA
 HvMpc2-BW   CACGTAGACGCGCTATTGTACAATGCTAAACGGGTCCACGTAGACGCGCTAATGACTAAATACGGCAACAACCCTGCTGCCCAAAGATGA
HvMpc2-DOM   CACGTAGACGCGCTATTGTACAATGCTAAACGGGTCCACGTAGACGCGCTAATGACTAAATACGGCAACAACCCTGCTGCCCAAAGATGA
HvMpc2-REC   CACGTAGACGCGCTATTGTACAATGCTAAACGGGTCCACGTAGACGCGCTAATGACTAAATACGGCAACAACCCTGCTGCCCAAAGATGA

             451                                                                  522
 HvMpc2-BA   CGGACCCTCACTCTCTCGCTCTCTAACACAACACAACACACACCGGAGAAGAGCGATAGAGAGAAGGAGAGA
 HvMpc2-BW   CGGACCCTCACTCTCTCGCTCTCTAACACAACACAACACACACCGGAGAAGAG--ATAGAGAGAAGGAGAGA
HvMpc2-DOM   CGGACCCTCACTCTCTCGCTCTCTAACACAACACAACACACACCGGAGAAGAG--ATAGAGAGAAGGAGAGA
HvMpc2-REC   CGGACCCTCACTCTCTCGCTCTCTAACACAACACAACACACACCGGAGAAGAG--ATAGAGAGAAGGAGAGA
```
