# Supplementary material for: Identification and characterization of regulatory network components for anthocyanin synthesis in barley aleurone
Source: BMC Plant Biol. 2017 Nov 14;17(Suppl 1):184. doi: 10.1186/s12870-017-1122-3 (PMC5688479; doi:10.1186/s12870-017-1122-3)

Additional file 3. The results of genotyping of the mapping population (Oregon Wolfe Barleys, OWB) for molecular mapping of barley genes: A – *HvMyc2* B – *HvMpc2* C – *HvF3’5’H*. Amplified fragments of the mapping population individuals of were digested with restriction endonucleases *Bse1* I, *Hga* I and *EcoR* I respectively (CAPS–analysis). DOM and REC are the parental lines.

A

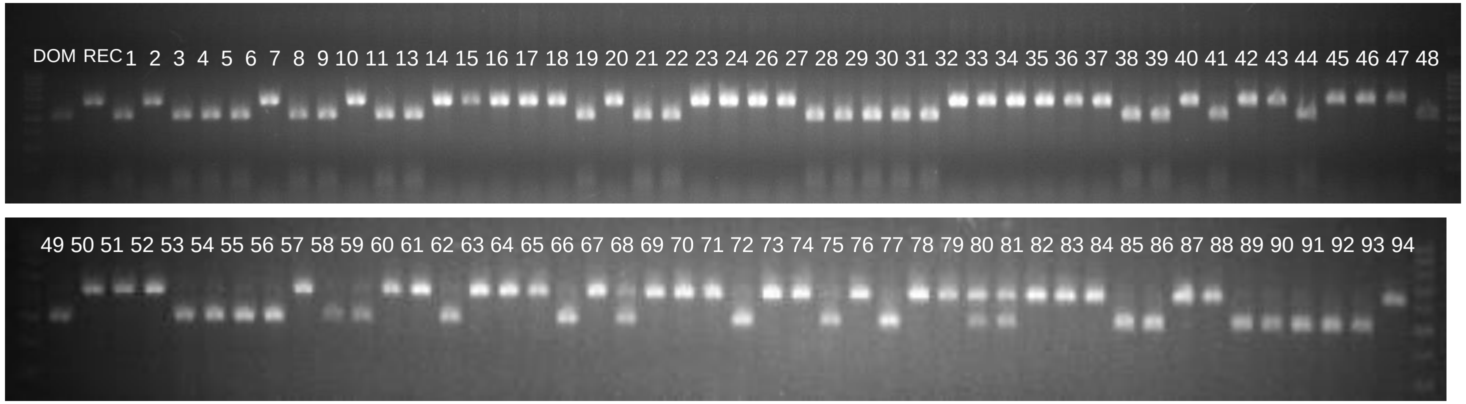

B

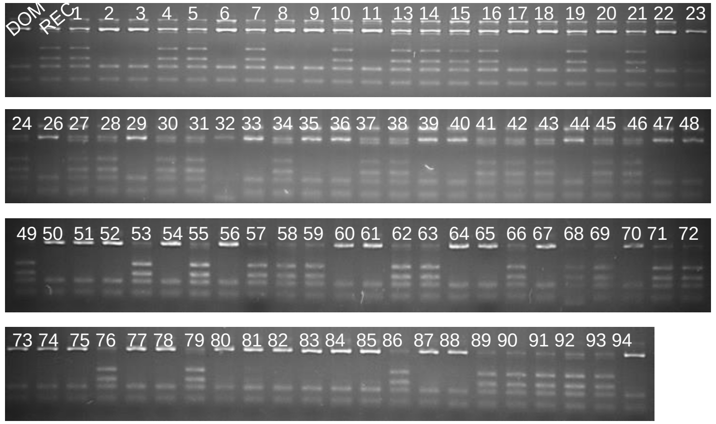

C

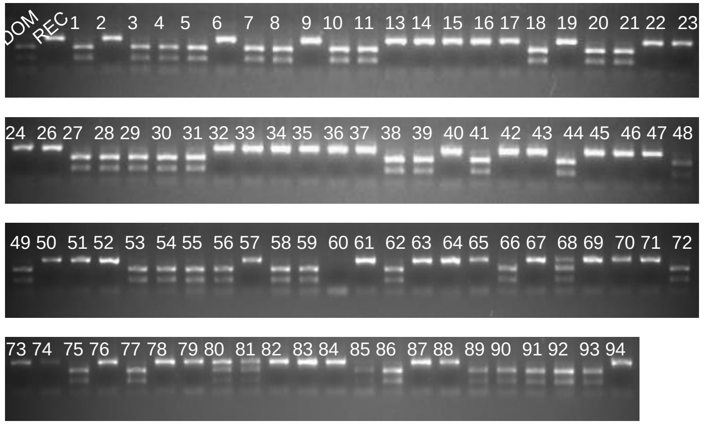

Supplement: Supplementary file 3 — The results of genotyping of the mapping population (Oregon Wolfe Barleys, OWB) for molecular mapping of barley genes: A – HvMyc2 B – HvMpc2 C – HvF3’5’H. Amplified fragments of the mapping population individuals of were digested with restriction endonucleases Bse1 I, Hga I and EcoR I respectively (CAPS–analysis). DOM and REC are the parental lines. (PDF 327 kb) [file 12870_2017_1122_MOESM3_ESM.pdf]
